# Establishment and Characterization of a Novel Gill Cell Line, LG-1, from Atlantic Lumpfish (*Cyclopterus lumpus* L.)

**DOI:** 10.3390/cells10092442

**Published:** 2021-09-16

**Authors:** Hilde Sindre, Mona C. Gjessing, Johanna Hol Fosse, Lene C. Hermansen, Inger Böckerman, Marit M. Amundsen, Maria K. Dahle, Anita Solhaug

**Affiliations:** 1 Norwegian Veterinary Institute, 1433 Ås, Norway; mona.gjessing@vetinst.no (M.C.G.); Johanna.Hol.Fosse@vetinst.no (J.H.F.); ingermarita.bockerman@fhi.no (I.B.); Marit.Masoy.Amundsen@vetinst.no (M.M.A.); maria.dahle@vetinst.no (M.K.D.); anita.solhaug@vetinst.no (A.S.); 2 Imaging Centre, Norwegian University of Life Sciences, 1432 Ås, Norway; lenececilie.hermansen@nmbu.no; 3 Norwegian Institute of Public Health, 0213 Oslo, Norway

**Keywords:** cleaner fish, lumpfish, respiration, epithelial, endothelial, virus, animal welfare, 3R, medical delousing, marine fish

## Abstract

The use of lumpfish (*Cyclopterus lumpus*) as a cleaner fish to fight sea lice infestation in farmed Atlantic salmon has become increasingly common. Still, tools to increase our knowledge about lumpfish biology are lacking. Here, we successfully established and characterized the first Lumpfish Gill cell line (LG-1). LG-1 are adherent, homogenous and have a flat, stretched-out and almost transparent appearance. Transmission electron microscopy revealed cellular protrusions and desmosome-like structures that, together with their ability to generate a transcellular epithelial/endothelial resistance, suggest an epithelial or endothelial cell type. Furthermore, the cells exert Cytochrome P450 1A activity. LG-1 supported the propagation of several viruses that may lead to severe infectious diseases with high mortalities in fish farming, including viral hemorrhagic septicemia virus (VHSV) and infectious hematopoietic necrosis virus (IHNV). Altogether, our data indicate that the LG-1 cell line originates from an epithelial or endothelial cell type and will be a valuable in vitro research tool to study gill cell function as well as host-pathogen interactions in lumpfish.

## 1. Introduction

Infestation with salmon lice (*Lepeophtheirus salmonis*) is one of the biggest challenges in farming of Atlantic salmon (*Salmo salar* L.). For years, bath treatment with anti-parasitic pharmaceuticals was the predominant treatment strategy. However, in the course of the past decades, a biological approach using cleaner fish feeding on lice from infested salmon has become more and more common. At present, Atlantic lumpfish (*Cyclopterus lumpus* L.) is the most commonly used cleaner fish species in Norwegian aquaculture, with about 43 million lumpfish used in 2019 [1]. Lumpfish is considered particularly suitable for this use, as they continue feeding at low temperatures and have a relatively short life cycle that allows them to be introduced into salmon farms already 4 months after hatching [2,3].

The extensive use of cleaner fish in salmon farming is in its infancy and is facing several challenges. A large proportion of cleaner fish die in the course of the production cycle [4]. Yet, we have a limited understanding of the causes of this mortality, in contrast to the knowledge we have of other farmed fish, like the salmon. The farming conditions are designed for salmon production, and even if environmental enrichments are provided, the conditions represent a suboptimal environment for lumpfish, with health and welfare issues as a consequence. Increased knowledge is needed to be able to provide the best conditions for salmon and lumpfish cohabitation. The most severe welfare issues in cleaner fish today are related to handling stress and infectious diseases [5]. The extensive cohabitation of two fish species at high densities is a biosecurity risk factor and facilitates the transmission of potential pathogens. Cross-species infections may pave the way for more virulent variants by increasing the chance of exposure and adaptation [6,7]. Outbreaks of the notifiable disease viral haemorrhagic septicaemia (VHS) have been reported in farmed lumpfish in Iceland [8], and a ranavirus similar to epizootic hematopoietic necrosis virus (EHNV) has been detected in lumpfish in Ireland, the Faroe Islands, Scotland and Iceland [9]. *Cyclopterus lumpus* virus (CLuV) was described in Norway in 2017 [10] and is associated with disease, severe liver lesions and high mortalities in lumpfish. A broad range of parasites have been identified in lumpfish, including both endo- and ectoparasites [7]. One of the latter is *Paramoeba perurans*, the causative agent of amoebic gill disease, leading to similar gill lesions as reported for Atlantic salmon [11]. Altogether, it is crucial to gain a better understanding of which pathogens can be harboured on cleaner fish in order to design screening programs to plot the infection status of both species.

Fish cell lines are valuable tools for in vitro fish research and may serve as models to mimic complex in vivo biology. A repertoire of cell lines from the species and organ of interest allows controlled and reproducible experimental conditions and may replace or reduce the number of experimental animals in line with the “3Rs” principles [12,13]. Fish cell lines serve as tools to study host cell-pathogen interactions, and for the isolation and characterization of viruses [14]. Furthermore, they have been extensively used to study cellular responses and the prediction of acute toxicity as a part of the hazard assessment of chemicals [15,16]. They can also be used to identify biomarkers as indicators of environmental pollutants, infection and disease [17].

The main function of gills is respiration. However, the gills also carry out other important processes, including osmoregulation, the excretion of nitrogenous waste and immunological functions. Hence, gill diseases may lead to compromise on several physiological levels. Because most gill diseases are not notifiable, the true extent and economic loss in aquaculture connected to compromised gill function is not known [18]. Only a few cell lines from gills have been established and published. One of them is the widely used gill epithelial cell line from rainbow trout (*Oncorhynchus mykiss*), RTgill-W1 [19]. Another gill epithelial cell line from Atlantic salmon, ASG10, was recently established in our lab [20]. Salmonids have a long evolutionary distance to lumpfish [21]; hence, cell lines from Atlantic salmon and rainbow trout are not reliable tools to study aspects of lumpfish biology. Apart from a fibroblastic cell line from lumpfish fin, characterized in 1977 [22], no cell lines from lumpfish have been reported.

In this study, we have addressed this gap and report the development and characterization of a novel gill cell line from lumpfish (LG-1). In the long term, our ambition is that LG-1 may serve as a tool to gain more insight into lumpfish biology and generate knowledge that can lead to a more robust lumpfish population in aquaculture.

## 2. Materials and Methods

### 2.1. Animal Husbandry and Ethical Considerations

Gills for the development of primary cells were obtained from an adult farmed lumpfish cultivated at a Norwegian commercial lumpfish farm. The fish was euthanized with an overdose of MS222 (Sigma-Aldrich, St-Louis, MO, USA. Two whole gill arches were then removed and incubated in medium consisting of Leibovitz-15 (L-15, Lonza, Basel, Switzerland) culture media with 10,000 units/mL penicillin, 10.0 mg/mL streptomycin (1% Pen/Strep; Lonza), 1 µg/mL Amphotericin B (Thermo Fisher Scientific, Waltham, MA, USA) and 20% fetal bovine serum (FBS superior, Biochrom, Cambridge, UK) and processed as described in Section 2.2 on the same day the tissue was harvested. For transmission electron microscopy, another fish was euthanized and the gills were processed as described in Section 2.7.

### 2.2. Development of Primary Cells

**Figure 1:**
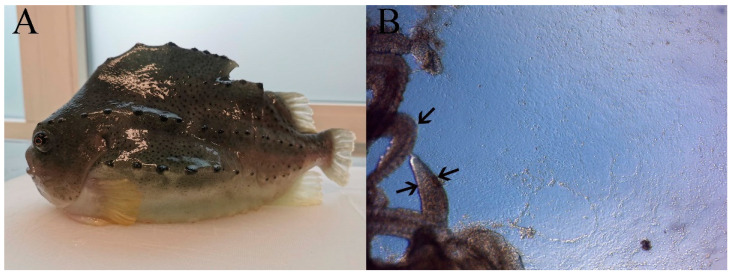
Lumpfish (**A**) and explants (**B**) from lumpfish gills, with gill filaments (left, indicated by arrows), an extensive growth of cells from the explant (middle and right part) and a confluent layer of cells (4 days after seeding). The thickness of the gill filament indicated by the arrows is about 200 µm. Microscope: Leica DMIL.

The gill arches were incubated twice for 10 min in a sterile 50 mL tube containing 40 mL Hanks’ balanced salt solution (HBSS, Lonza) supplemented with 0.05 mg/mL gentamycin (Lonza) with constant gentle rotation (30–60 rpm). The gill arch was then placed in a sterile petri dish and the cartilage of the arch was removed. A few droplets of supplemented L-15 medium: L-15 (Lonza) with an addition of 0.1 mM of non-essential amino acid, (Lonza), 1 mM sodium-pyruvate (Lonza), 0.01 mg/mL insulin, 0.01 mg/mL transferrin and 0.01 µg/mL selenium (ITS, Lonza), 4 mM L-glutamine (Lonza), 0.05 mg/mL gentamycin (Lonza), 0.03 mM 2-mercaptoethanol (Gibco^™^, Thermo Fisher) and 20% FBS, were added to the gill filaments. The filaments were cut by a scalpel in explants of 1–2 mm and about 2–4 explants were plated in 25 cm^2^ cell bind flasks (Corning, New York, NY, USA) with 1 mL of supplemented L-15 medium to allow the tissue pieces to adhere. After one day, when the explants had adhered to the surface of the flasks, 2 mL of supplemented L-15 medium were gently added. Then, 2 days later, adherent cells were confirmed under the micropscope and the medium was changed to remove gill residuals and dying cells. The cells were then grown further at 15 °C for 4 weeks until a confluent monolayer was established (Figure 1B). The cells were then detached as followed: the cells were washed once with 5 mL PBS and 0.5 mL 0.25% trypsin/EDTA (Lonza) were added to the cells. After 10–15 min in room temperature, the cells detached and were passaged 1:2 to new cell culture flasks in 5 mL supplemented L-15 medium. The procedure was repeated several times until stable dividing cell cultures were established. In passage 7, the cell culture was tested negative for mycoplasma (Myco Alert; Lonza).

### 2.3. Routine Maintenance

The cells were maintained in complete cell culture medium: Leibovitz’s L-15 Medium, GlutaMAX™ Supplement (Gibco™, Thermo Fisher) with an addition of 10% FBS, 100 units potassium penicillin and 100 μg streptomycin sulfate (1% pen/strep, Lonza). The cells were cultured at 20 °C, which gives a proliferation rate that allows for sub-cultivation 1:2 every second week. This is done as followed: the cells were washed with 10 mL PBS, and 2 mL 0.25% trypsin/EDTA were added to the cells. After 10–15 min in room temperature, the cells detach, then 10 mL of complete cell culture medium were added. 6 mL of the cell suspension, containing about 2.5 × 10^6^ cells, were then transferred to a new 75 cm^2^ flask. One confluent flask (75 cm^2^) contains about 5.0 × 10^6^ cells. For experiments, passage number 15–40 was used. No differences were observed between early and late passage numbers with regard to proliferation or morphology. The cells were seeded on standard plastic cell culture plates, 100,000 cells/cm^2^, resulting in a confluent cell layer the next day. The LG-1 cells can successfully be stored by cryopreservation. Here, 5 × 10^6^ cells were suspended in 2.5 mL FBS. This cell suspension was then transferred to 1.8 mL Cryo Tube^™^ Vials (Nunc^™^, Thermo Fisher), 0.5 mL/tube, and then 0.5 mL freezing medium (L-15 medium, 1% Pen/Strep, 10% FBS, 20% DMSO) were gently added to each tube. The cells were then placed in a Mr Frosty freezing container (Nalgene™, Thermo Fisher) at −80 °C, which allows for a gradual reduction in temperature. After 2 days, the cells were transferred to liquid N_2_ for continued storage (−196 °C). For shorter storage periods, the cells (a confluent flask) can also be stored in the refrigerator at 4 °C. At this temperature, some cells will die, but after a short period at 15–20 °C, the remaining cells will start proliferating again.

### 2.4. Species Identification

RNA was extracted using RNeasy Mini kit (Qiagen, Hilden, Germany), following the manufacturers protocol. Briefly, 600 µL RLT lysis buffer (Qiagen) and 6 µL β-mercaptoethanol (<99%) were used to lyse LG-1 cells in a 75 cm^2^ cell culture flask. The lysate was homogenized using a 1 mL syringe. The quantity and purity of the extracted RNA were measured using a NanoDrop™ 2000 spectrophotometer (Thermo Scientific). For transcription analysis, cDNA was synthesized using 1000 ng of total RNA using a QuantiTect Reverse Transcription kit (Qiagen) with gDNA elimination, according to the manufacturer’s instruction. The samples were incubated for 30 min at 42 °C to activate reverse transcription and then for three min at 95 °C to inactivate the reaction. After synthesis, the samples were frozen and stored at −20 °C. The qPCR was performed in duplicates with 5 ng of cDNA input in a total volume of 10 µL per reaction using SsoAdvanced™ Universal SYBR^®^ Green Supermix (Bio-Rad, Hercules, CA, USA). The thermal program was set to 95 °C for 30 s, 39 cycles of 95 °C for 15 s and 60 °C for 30 s. Primers for Atlantic lumpfish (*Cyclopterus lumpus* L.) interleukin 6 (IL-6) were designed using NCBI primer Blast™ [23], and levels of Atlantic lumpfish (*Cyclopterus lumpus* L.) interleukin 6 (IL-6) and elongation factor 1 α (EF1α) [24] were assessed using 10 µM primers. Primers targeting Atlantic salmon *Salmo salar*) Elongation factor 1α (ss-EF1a) [25] were used as a negative control. Amplicon length of each qPCR product was controlled using a 2100 Bioanalyzer (Agilent Technologies, Santa Clara, CA, USA) along with an associated kit, Agilent DNA 1000, according to the manufacturer’s protocol. The primers used are shown in Table 1.

### 2.5. Proliferation

The cells were seeded in a 96 well plate, 50,000 cells/cm^2^, and cultured for 1–14 days at different temperatures (20, 16, 10, 4 °C). For quantification, the cells were stained with DRAQ5 (Thermo Fisher; nuclear staining, 1:500) for 30 min at room temperature and the cell number in a specific area of the well was counted by the spectramax i3x plate reader equipped with a microscopic module (MiniMax300Imaging Cytometer, Molecular Devices, San Jose, CA, USA).

### 2.6. Morphology

The cells were seeded in 6-well plates (100,000 cells/cm^2^). At confluence, the cells were stained with Calcein-AM (1 µM; Sigma) and visualized by light and fluorescence microscopy (Axio Observer A1, Zeiss, Jena, Germany).

### 2.7. Transmission Electron Microscopy (TEM)

Lumpfish gills (from 2.1) were cut into 1 mm^2^ under fixative (2% paraformaldehyde/1.25% glutaraldehyde/0.1 M cacodylate buffer) and stored at 4 °C until further processing, as described for the ASG10 cells [20]. The LG-1 cells were seeded on transwell inserts (Costar™ 0.4 µm polyester membrane, Sigma-Aldrich) or in a culture flask, 100,000 cells/cm^2^. After 5 days, the cells were washed once with PBS, the cells on the membrane were fixed directly on the membrane and the cells in the flask were scraped and pelleted by centrifugation (500× *g*, 10 min) prior to fixation. Fixation were done in 2% paraformaldehyde/1.25% glutaraldehyde/0.1 M cacodylate buffer for 15 min at room temperature, washed with 0.1 M sodium cacodylate buffer, embedded in 3% low-melting agarose and post-fixed in 1% osmium tetroxide in 0.1 M sodium cacodylate buffer for 1 h. Subsequently, the cells were washed thoroughly in 0.1 M sodium cacodylate buffer, dehydrated with 10 min steps in ascending ethanol series (50–100%) and embedded in LR White resin (London Resin Company, EMS, Agar Scientific, Stansted, UK). Ultrathin sections were obtained using a Leica EM UC6 Ultramicrotome (Leica, Wetzlar, Germany). The sections were stained with 4% uranyl acetate and 1% potassium permanganate for 10 min and examined and photographed using a FEI Morgagni 268 transmission electron microscope (FEI, Hillsboro, OR, USA). Contrast and white balance were adjusted in Adobe Photoshop software (Adobe systems, San Jose, CA, USA).

### 2.8. Periodic Acid-Schiff (PAS), Alcian Blue pH 1 Staining and Immunostaining for Chloride Cells

The cells were fixed for 10 min in 4% paraformaldehyde at room temperature, washed with PBS, stained with PAS and Alcian blue for the detection of mucus cells as described in [26] and immunostained for chloride cells as, as previously described In short, the cells were washed gently in PBS, incubated for 20 min in Tris-buffered saline (TBS) with 2.5% bovine serum albumin (BSA) for prevention of non-specific binding, incubated at 60 min with primary antibody directed against a conserved region of Na^+^/K^+^ ATPase subunit (a5, Developmental Studied Hybridoma Bank, University of Iowa, Iowa City, IA, USA and diluted 1:100 in TBS with 2.5% BSA. A biotinylated rabbit anti mouse secondary antibody and an alkaline phosphatase conjugated streptavidin system was used to visualize the binding. Paraffin embedded gills were used as positive controls and inspected under a Leica microsope.

### 2.9. Immunostaining for Cell Markers

The cells were plated on Millicell EZ slides (Merck, Darmstadt, Germany). At confluence, the cells were washed once in PBS and fixed with 4% paraformaldehyde for 15 min at room temperature. For F-actin (phalloidin) staining, the cells were permeabilized with saponin (0.05%) for 10 min at room temperature and stained with phalloidin Alexa fluor 555 (#8953, 1:300, Thermo Fisher) for 30 min. The cells were then washed 3 times with PBS and coverslips mounted with prolong mounting medium. Confocal fluorescence microscopy was performed using a Zeiss LSM710 microscope. For recombinant mouse anti-pan cytokeratin (clone [C-11] (ab7753), 1:1000, Abcam, Cambridge, UK), purified mouse anti-E-Cadherin (clone 36/E-Cadherin, 1:1000, BD Biosciences, Franklin Lakes, NJ, USA), mouse anti-human ZO-1 (#33-9100, clone ZO1-1A12, 1:100, Thermo Fisher) and rabbit polyclonal anti-human von Willebrand factor (DAKO A0082, 1:1000, Agilent) staining, the cells were seeded and fixed as described above and permeabilized in ice cold methanol (100%). The cells were then washed 3 times with PBS and blocked in 5% BSA for 60 min followed by incubation with primary antibodies overnight at 4 °C. The cells were then rinsed 3 times and incubated with secondary antibody conjugated with Alexa Fluor 488 (Molecular probes, Thermo Fisher) for 2 h at room temperature. The cells were then washed 3 times with PBS, the nuclei were stained with DAPI (1:1000) and coverslips were mounted with a prolong mounting medium. Confocal fluorescence microscopy was performed using a Zeiss LSM710 microscope.

### 2.10. Transepithelial/Transendothelial Electrical Resistance (TER)

The cells were seeded on transwell inserts (Costar 0.4 µm polyester membrane; 100,000 cells/cm^2^) and TER was automatically measured every 6 h, using CellZscope E (Nano Analytics, Münster, Germany).

### 2.11. CYP1A Activity

The induction of CYP1A is measured by the EROD-assay in the presence of NADPH (β-nicotinamide adenine dinucleotide phosphate). CYP1A converts the artificial substrate EROD to resorufin, which can be measured via fluorescence spectroscopy. The assay was done as described in [27] with minor modifications. Briefly, the cells were seeded in black 96 well plates (100,000 cells/cm^2^) and incubated for 24 h in culture medium. The cells were then incubated with the CYP1A inducer, beta-naphtaflavone (BNF; 1–100 nM; Sigma-Aldrich) for 24 h. The next day, the culture medium was replaced with 200 µL EROD assay media (DMEM w/o phenol red; Gibco™, Thermo Fisher, 10% FBS, 8 µM 7-Ethoxyresorufin; Sigma-Aldrich). After 30 min of incubation, resorufin fluorescence (Ex: 530 nm/Em: 580 nm) was quantified using a plate reader (Spectramax i3x plate reader, San Jose, CA, USA).

### 2.12. Testing for Susceptibility to Fish Viruses

To test for permissiveness to different viruses, LG-1 were incubated with viruses, as listed in Table 2 [28,29]. In short, the cells were seeded in 96 well plates (Corning^®^) using complete cell culture medium containing 15% FBS and grown to approx. 80–90%. The growth medium was then removed and 50 µL of complete growth medium without FBS containing about 50 TCID_50_ of each virus were added. Following 3–4 h of incubation at 15 °C, 150 µL of complete growth medium containing 10% FBS were added to each well. In addition, negative controls without the virus were included. The plates were incubated at 15 °C for 7 days for IHNV, IPNV, betanodavirus, ISAV and SAV1, 2 and 3. The incubation of the plate inoculated with VHSV was terminated at full CPE 4 days post infection. The cells and supernatants from wells inoculated with CLuV, were harvested 7, 14 and 21 days post-inoculation and tested for virus propagation by RT-qPCR, as described in [10]. For the other viruses, the medium was removed from all wells and IFAT was performed as described previously [30]. In short, the cells were fixed with 80% acetone for 20 min, dried and stained with specific antibodies against the individual viruses as listed in Table 2 [31,32], and biotin labelled goat anti-mouse Ig (E 0433, Dako) and FITC-labelled streptavidin (11-4317, AH diagnostics, Tilst, Denmark) were used for the secondary and tertiary step, respectively. For the staining of betanodavirus, FITC-labeled goat anti-rabbit IgG (4030-02, Southern Biotech, Birmingham, AL, USA) was used as secondary antibody. The nuclei were visualized by staining with propidium iodide (Sigma-Aldrich). The stained preparations were evaluated by wide field inverted fluorescence microscopy (Leica DMIL).

For further investigation into susceptibility to SAV1, nodavirus, IHNV and VHSV, LG-1 and OIE-recommended cell lines for each virus type were seeded in 25 cm^2^ flasks. At about 80%-confluence LG-1 was inoculated with 500 TCID_50_ of SAV1, fish nodavirus, IHNV and VHSV, whereas OIE-recommended CHSE-214 (ATCC CRL-1681), E-11 (ECACC 01110916), EPC (ATCC CRL-2872) and BF-2 (ATCC CCL-91) were inoculated with the same amounts of SAV1, nodavirus, IHNV and VHSV, respectively. Following 14 days of incubation at 15 °C (except for E-11, which was incubated at 20 °C), the supernatant was harvested. The virus titration of cell supernatants following virus propagation was performed in 10-fold dilution series in 6 parallel wells with an incubation period of 7 days. The cells were then fixed and IFAT performed, as described above. A TCID_50_ titer was then calculated according to the Spearman-Kärber method [33].

### 2.13. Statistical Analysis

The data analyses were performed using Sigma Plot version 12.0 (Systat Software, San Jose, CA, USA). Statistical significance (*p* < 0.05) was assessed using 1-way-ANOVA, followed by Dunnetts post-test. For analysis of cell proliferation data in 3.2, Normality test (Sharpiro-Wiik) failed. Kruskal-Wallis One Way Analysis of Variance on Ranks followed by Dunnetts post-test was therefore used.

## 3. Results

### 3.1. Development and Maintenance of the LG-1 Cell Line

**Figure 2:**
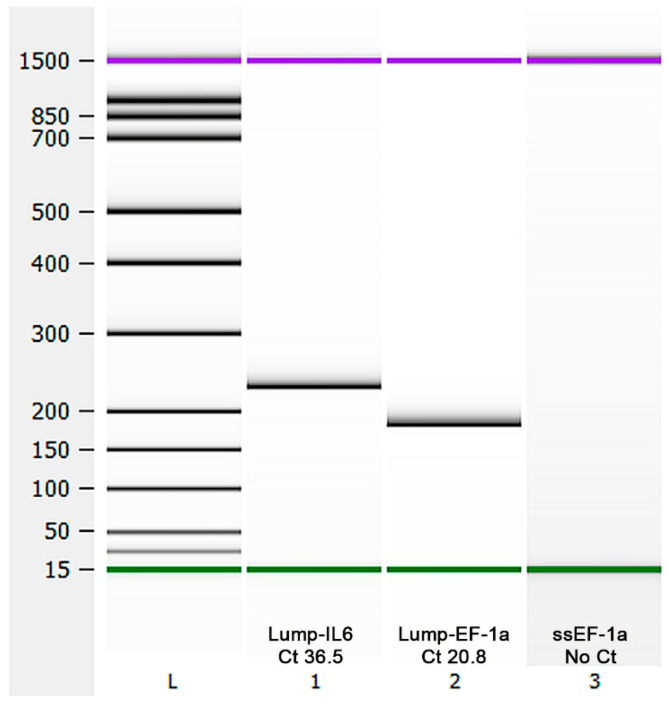
Control of lumpfish origin. The origin of the LG-1 cells was controlled by RT-qPCR on total RNA using lumpfish-specific primers targeting IL-6 and EF1α, and Atlantic salmon specific primers targeting EF1α were used as a negative control. The correct amplicon length was confirmed by capillary electrophoresis (Bioanalyzer). Lump: Lumpfish. Ss: *Salmo salar*.

A primary cell line (LG-1) was established from lumpfish gill explants as illustrated in Figure 1B. The species origin of the LG-1 cell line was determined by RT-qPCR using species-specific primers targeting the housekeeping mRNA encoding Elongation factor 1a from lumpfish (LumpEF1a) and Atlantic salmon (ssEF-1a) as negative control, as well as lumpfish interleukin 6 (LumpIL-6), a cytokine gene expressed by epithelial and endothelial cells [34,35]. Both lumpfish mRNAs, but not the Atlantic salmon housekeeping mRNA, were detected in the sample. The amplicon products were confirmed to be of correct length, according to the target sequences (Figure 2).

### 3.2. Proliferation

**Figure 3:**
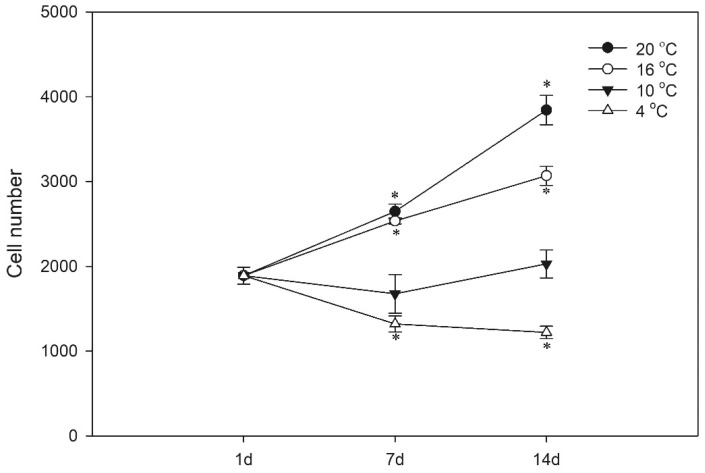
Cell proliferation. The cells were plated at different densities and grown for 1, 7 and 14 days (d) at different temperatures, as indicated. The data are representative of three independent experiments and are expressed as mean ± SD of four parallel incubations. * indicates significantly different from day 1.

To define optimal growth conditions for the LG-1 cells, we evaluated cell proliferation at different temperatures. We seeded cells at a density (50,000 cells/cm^2^) that resulted in approximately 50% confluence after 1 day. After 1, 7 and 14 days, the cells were stained with the nuclear stain DRAQ5 and counted by a plate reader equipped with an imager module (Figure 3). When grown at 20 °C, cell numbers doubled within 14 days. At 16 °C, the cells grew at similar rates as at 20 °C for the first 7 days but then reduced their proliferation rate. At 10 °C, no change in cell numbers was observed at any time point, and at 4 °C, cell numbers declined, probably due to cell death, from days 1 to 7, but remained constant between days 7 and 14.

### 3.3. Cell Morphology

**Figure 4:**
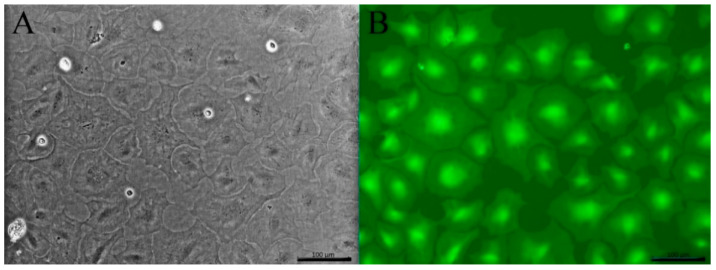
Cell morphology of the established LG-1 cell line. (**A**) Light microscopy. (**B**) The cells were stained with the live cell stain Calcein-AM and visualized by fluorescence microscopy. Scale bar = 100 µm Microscope: Leica DMIL.

**Figure 5:**
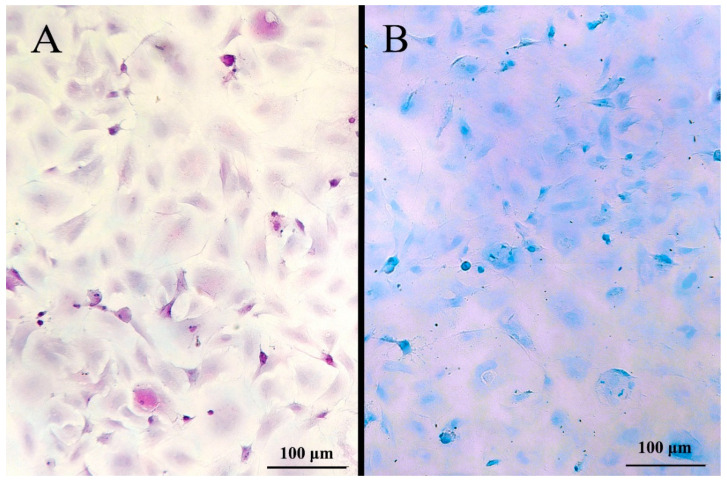
LG-1 cells stained with PAS (**A**) and Alcian blue (**B**) to clarify the presence of mucus cells (bright pink or blue, respectively). Microscope: Leica DMIL.

When examined by light microscopy, the adherent LG-1 cell population appeared homogenous with a flat, polygonal and stretched-out, almost transparent, appearance (Figure 4A). Flow cytometry supported the observation of a homogenous cell population, as the cells clustered on a side scatter forward scatter plot, suggesting cells of similar size and complexity (data not shown). Staining the cells with the live cell stain Calcein-AM made it easier to assess their morphology (Figure 4B). When reaching confluence, the cells stopped proliferating (data not shown), demonstrating that LG-1 cells are responsive to contact inhibition. Confluent cultures remained viable at 20 °C for at least 3 weeks (data not shown). PAS and Alcian blue staining suggested that no goblet cells were present in the LG-1 cultures (Figure 5). Similarly, staining for ATPase gave no signal, suggesting that chloride cells are not present in the cell population (data not shown).

**Figure 6:**
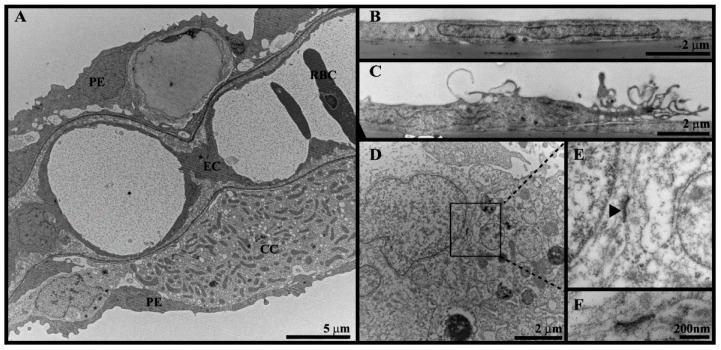
Ultrastructure of whole lumpfish gill (**A**) and LG-1 cells (**B**–**F**). (**A**) TEM of gill showed pavement epithelial cells (PE), pillar endothelial cells (EC), red blood cells (RBC) and chloride cells (CC). (**B**–**F**) TEM of LG-1 cells showed (**B**) elongated shape, (**C**) surface protrusions and (**D**–**F**) structures compatible with desmosomes.

Transmission electron microscopy was performed to further evaluate the morphological features of the LG-1 cells (Figure 5). To our knowledge, the ultrastructural features of lumpfish gill cells have not been described, and we therefore included whole gill tissue as a reference (Figure 6A). Resembling observations in rainbow trout [19], the surface of lumpfish gill lamella was covered by thin, elongated pavement epithelial cells with a ruffled surface (Figure 6A, PE). Pillar endothelial cells covered the lumen of the gill lacuna (Figure 6A, EC). We also observed red blood cells (Figure 6A, RBC), a mitochondrial-rich cell, often referred to as chloride cells (Figure 6A, CC), and cells compatible with goblet cells (data not shown). The TEM analysis of LG-1 confirmed the flattened shape observed by wide field microscopy (Figure 6B,C). In line with the results from specific stains described above, the features of LG-1 cells were clearly distinct from the characteristic chloride cells and the mucus-containing goblet cells observed in tissue sections. Interestingly, some cells formed surface protrusions (Figure 6C). Altogether, the TEM observations are compatible with properties of the flat pavement epithelium observed in the whole lumpfish gill, but a pillar endothelial cell type cannot be excluded. Moreover, a few desmosome-like structures were seen (Figure 6D–F), further indicating that the LG-1 cell line is of epithelial or endothelial origin.

**Figure 7:**
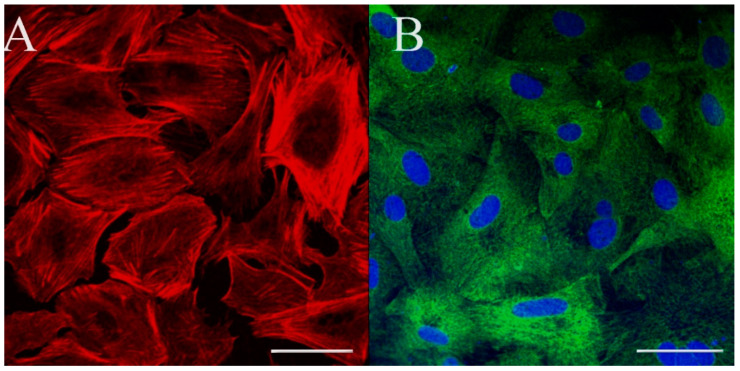
Cytoskeletal filament profile of the LG-1 cells. F-actin (**A**; phalloidin; red) and cytokeratin (**B**; green) were stained and visualized by confocal microscopy. Scale bar = 50 µm.

Phalloidin, which stains filamentous actin, revealed stress fibers and circumferential actin filaments (Figure 7A). The cells also exhibited positive staining for cytokeratin, further supporting an epithelial or endothelial cell type (Figure 7B). No specific signal was observed when staining for the E-cadherin, ZO-1 or the endothelial-specific von Willebrand factor (data not shown). Notably, these antibodies are raised against human peptides and while they, in our experience, cross-react with epitopes of Atlantic salmon proteins, their ability to recognize lumpfish proteins remains unknown. As a result, the significance of these negative findings remains unclear.

### 3.4. Generation of TER

**Figure 8:**
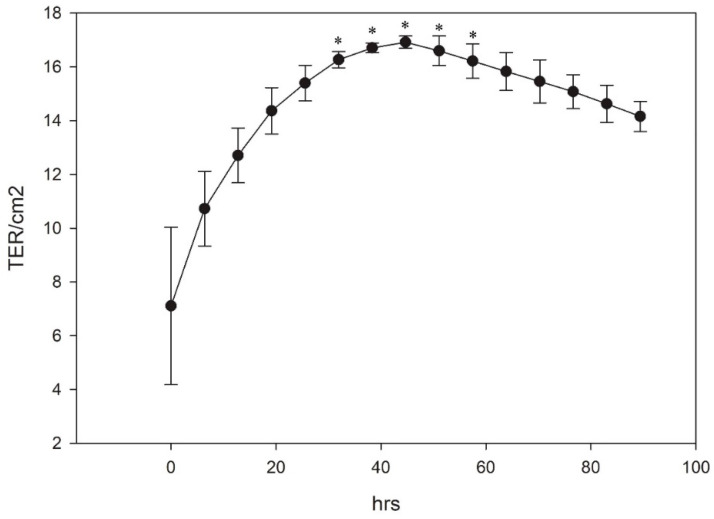
The cells were plated on transwell membranes and TER values measured every 6 h. The results represent mean ± SEM of 3 independent experiments. * indicates significantly different from 0 h.

The formation of a selective permeable barrier is an important functional characteristic of both epithelial and endothelial cells. Transepithelial/transendothelial electrical resistance (TER) is a widely accepted quantitative measurement of the integrity of a cellular monolayer and tight junction dynamics [36]. To evaluate the ability of the LG-1 cell line to generate TER, the cells were seeded on transwell membranes and TER was measured every 6 h over a period of 4 days (96 h). The TER increased over a period of 48 h when it reached its peak at about 16 TER/cm^2^ (Figure 8). After this, the TER gradually decreased. When cells were cultured for a longer period, the TER continued to decline, reaching approximately 8 TER/cm^2^ at 256 h (10 days, data not shown).

### 3.5. CYP1A Induction

**Figure 9:**
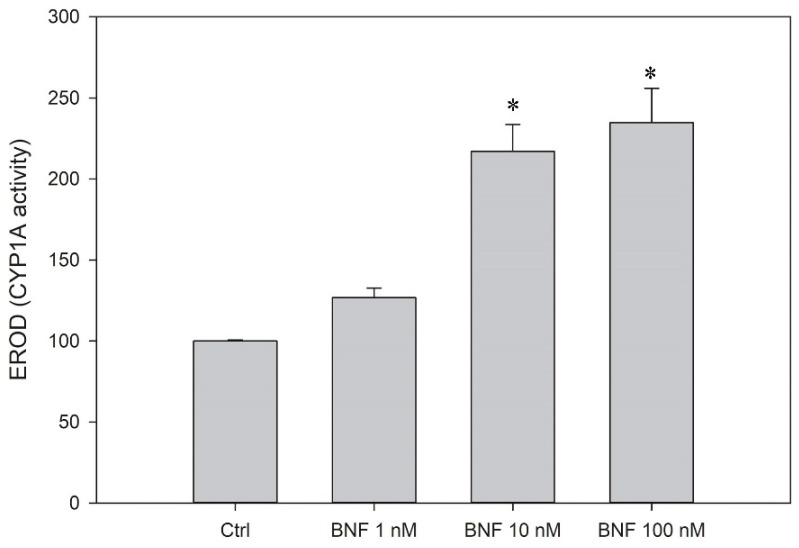
CYP1A induction. The cells were treated with BNF for 24 h and their catalytic activity was measured by the EROD assay. The results represent mean ± SEM of 3 independent experiments. * indicates significantly different from control.

Both gill epithelial cells and pillar endothelial cells are able to upregulate and activate CYP1A, a key enzyme in oxidative metabolism, in response to the stimulation of the aryl hydrocarbon receptor. To investigate the ability of the LG-1 cells to induce CYP1A activity, the cells were treated with beta-napthoflavone (BNF; 24 h), an aryl hydrocarbon receptor agonist and a known inducer of CYP1A expression [27]. The subsequent CYP1A activity was measured by using the EROD assay, and, here, the BNF-exposed LG-1 cells had a significant higher CYP1A activity (Figure 9).

### 3.6. Susceptibility to Fish Viruses

**Figure 10:**
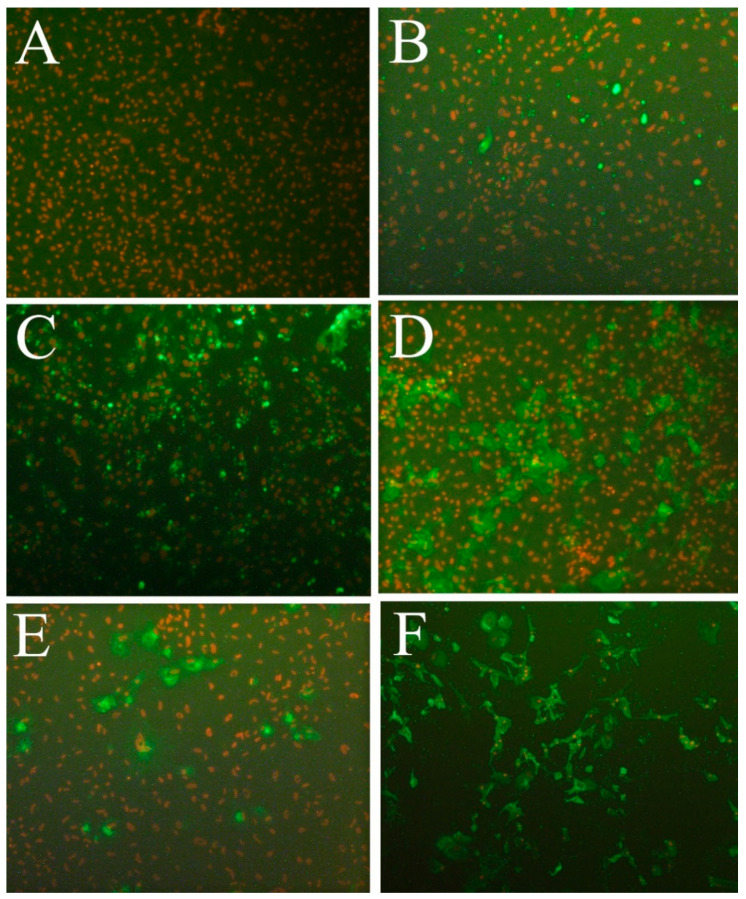
Susceptibility of LG-1 cells to fish viruses. LG-1 cells inoculated with (**A**) mock control, (**B**) ISAV, (**C**) betanodavirus (BFNNV genotype), (**D**) SAV1 (**E**) IHNV genogroup M and (**F**) VHSV genogroup III. Viral protein visualized by IFAT (green), cell nuclei by propidium Iodide (PI, red). Viral isolates and detection antibodies described in Table 2. Microscope: Leica DMIL.

The LG-1 cells were inoculated with a panel of viruses associated with severe disease in farmed fish. Following 4–7 days of infection, the cells were fixed and an IFAT performed to visualise virus uptake and propagation in the cells. LG-1 cells were not permissive to IPNV (sp serotype) or lumpfish flavivirus (data not shown). After infection with ISAV, SAV2 or SAV3, single cells stained positive for viral antigens, indicating either uptake of the virus or limited viral protein expression in individual cells (demonstrated for ISAV in Figure 10B). However, there was no observed CPE nor an increase in viral load over time and no further transmission of the virus after prolonged incubation (>21 days), indicating that LG-1 cells do not support the propagation of these viruses. In contrast, LG-1 cells were susceptible to infection by SAV1, fish nodavirus (BFNNV genotype), IHNV genogroup M and VHSV genogroup III, resulting in typical cytopathic effect (CPE) and the distinct cellular expression of viral antigens (Figure 10C–F). For VHSV, CPE developed very rapid and complete CPE was observed within a few days after inoculation. (Figure 10F). The susceptibility of LG-1 to SAV1, fish nodavirus, IHNV and VHSV was investigated further by comparing the viral titers obtained in LG-1 to titers obtained in OIE recommended standard cell lines used for propagation and diagnostic purposes (CHSE-214, E-11, EPC and BF-2, respectively). The propagation of VHSV in LG-1 cells resulted in a slightly higher titer than in BF-2, 10^6.8^ TCID_50_/mL and 10^6.3^ TCID_50_/mL, respectively. For the other viruses, titers obtained in LG-1 cells were >2 log lower than in standard cell lines.

## 4. Discussion

Here, we document the establishment of a stable gill cell line (LG-1), maintained for 40 passages and with confirmed lumpfish origin. The cell line was defined as an epithelial or endothelial cell type, based on several findings. First, the morphological features of LG-1 cells resemble those of squamous epithelium, as they are adherent, homogenous and have a flat, stretched-out, almost transparent appearance. Furthermore, the thin and delicate nature of LG-1 cells corresponds to our observations of pavement epithelial cells in this species and is consistent with the main function of the gills, namely respiration. Second, the LG-1 cells form a confluent monolayer with a cobble-stone appearance, and cell division is inhibited upon cell-cell contact/contact-inhibition. Third, LG-1 cells form close contacts, with structures consistent with desmosomes, the anatomical hallmark of barrier cells such as epithelial and endothelial cells, and they generate trans-epithelial resistance (TER). Interestingly, the TEM images also clearly showed that some LG-1 cells generated long cellular protrusions. Epithelial and endothelial cells also typically express IL-6 [34,35], as shown here using IL-6 qPCR.

Predicting acute toxicity in fish is important, and fish cell lines represent an attractive alternative method to using live fish. The gills are one of the major extra-hepatic sites of metabolic activity. Accordingly, the RTgill-W1 has been extensively used in research over the last decade, demonstrating a close correlation between RTgill-W1 cytotoxicity and acute fish toxicity [15,37]. The detoxification capabilities of the gills includes the phase I enzyme CYP1A, whose activity can be measured by the EROD assay. CYP1A activity is often used as a sensitive biomarker of exposure to organic and inorganic environmental pollutants with a planar structure, such as 2,3,7,8-Tetrachlorodibenzo-p-dioxin (TCDD), Benzo(a)pyrene (B(a)P), polychlorinated biphenyls (PCBs) and Polybrominated diphenyl ethers (PBDEs). To detect these chemicals, the EROD assay is used by several cellular ex vivo and in vitro systems, such as gill filaments [38], primary fish liver and gill cell cultures [39,40] and the rainbow trout liver cell line RTL-W1 [41]. Interestingly, the LG-1 cells can induce CYP1A activity and may thus serve as a tool to study biotransformation or as a biosensor. In conclusion, the LG-1 cell line may contribute to toxicity testing; however, its sensitivity towards known toxins should first be evaluated.

The isolation and propagation of infectious agents in cell culture are needed to allow for the identification and characterization of emerging viruses and their interaction with host cells. Cellular factors that regulate the permissiveness to infection and disease, including surface proteins used for viral attachment and intrinsic antiviral responses, generally differ between species. An example is the manifestation of amoebic gill disease, which in lumpfish is different from that in salmon, with a slower development of characteristic pathology [11], suggesting species-specific strategies to combat *Paramoeba perurans*. This difference may be related to aspects of the environment or agent, but putative species differences can now be investigated using gill cell lines from the different species. Hence, the LG-1 cell line is an important addition to the current diagnostic toolbox in aquaculture.

Many aquatic viruses replicate in fish gill epithelium and may pass through this thin cell layer, leading to systemic infection. LG-1 cells supported the growth of SAV1, the causative agent of pancreas disease in Atlantic salmon. SAV exists in six genotypes. Genotype SAV3 so far is exclusively found in Norway, marine SAV2 is found in both the UK and Norway and SAV1 has not been detected in Norway but is endemic to the UK and Ireland [42]. While SAV1 was propagated in LG-1, the cells were not permissive to either SAV2 or SAV3. This susceptibility of lumpfish to SAV1 is novel and highly interesting, as the marine reservoirs for the different SAV genotypes have not been identified. Although some SAV genotypes have been found in marine fish species like the common dab [43,44], pancreas disease is only described in salmonids [45].

Although nodavirus has not been detected in either farmed or wild-caught lumpfish so far, a previous challenge trial has shown that lumpfish can be experimentally infected. The fact that LG-1 supported the growth of a betanodavirus field isolate from Norwegian halibut indicates that gill cells from lumpfish express surface proteins that mediate nodavirus infection. In Norway, this virus has already been detected in connection with disease in farmed cod, halibut and turbot [30,46,47,48,49]. The introduction of nodavirus into lumpfish farms may be of concern, as the virus is known to cause persistent infections and disease in susceptible species. However, salmonids are not considered natural hosts for nodavirus infections [48], although the virus can be introduced experimentally [49]. Hence, the risk of natural transmission of the virus and associated disease to farmed salmon would probably be low, although the long-term cohabitation of species may increase possible transfer and adaptation, as noted earlier.

VHS is a notifiable disease in both EU and OIE, and the introduction of VHSV into Norwegian aquaculture may seriously impact both fish health and trade. The LG-1 cells supported the efficient propagation of a field isolate of VHSV genogroup III from an outbreak of VHS of presumed marine origin in a rainbow trout farm in Norway [38]. Outbreaks of VHS in farmed cleaner fish, including lumpfish, have already been reported in other countries [8,50], and the screening of wild fish populations has demonstrated that VHSV is present in marine fish reservoirs both in Scotland, Norway, Denmark and the Baltic Seas [51,52,53]. So far, disease caused by the virus has only been reported in rainbow trout in Norway, but close contact between salmon and lumpfish may facilitate an adaptation of virus and “host-jumping”, a strategy associated with many other RNA viruses like coronaviruses and influenza viruses [54,55].

In conclusion, LG-1 provides a new tool to study gill epithelial cell function as well as the detection, propagation and characterization of viruses from lumpfish and other marine species. Furthermore, the use of LG-1 as a biosensor for toxins and xenobiotic activity of CYP1A appears promising.

## Figures and Tables

**Table 1:** Primer over view; species identification.

Gene	Primers	Amplicon Length	Accession	References
*Cyclopterus lumpus* IL-6	Fwd: 5′-CAC CAT CAA CCA CAG ACG GA-3′ Rev: 5′-AAC GGC GCT TAC TGA GTT GA-3′	224	MN093127.1	Designed in house
*Cyclopterus lumpus* EF1a	Fwd: 5′-GGC CAG ATC AAT GCC GGA TA-3′ Rev: 5′-CTC CAC AAC CAT GGG CTT CT-3′	189	XM_034545962.1	[24]
*Salmo salar* EF1a	Fwd: 5′-TGC CCC TCC AGG ATG TCT AC-3′ Rev: 5′-TCA CCA GGC ATA GCC GAT TC-3′	175	XM_014141923.1	[25]

**Table 2:** Overview of virus isolates and primary antibodies.

Virus Isolate	Primary Antibody
ISAV Glesvaer AF404340	anti-ISAV P10, Aquatic Diagnostics, Glasgow, UK
SAV1 140699 SPDV WSV	Anti-SAV 17H23, [31]
SAV2 MR-R5-2011, HE863664	Anti-SAV 17H23, [31]
SAV3 R-1_2007, LT630447	Anti-SAV 17H23, [31]
VHSV genogroup III [28]	Anti-VHS, IP5B11, BIO-X Diagnostics, Rochefort, Belgium
IHNV genogroup M, LR-80, AY442514	050690 Mab G Protein, gift Oregon State University, USA
Betanodavirus, AH95NorA (BFNNV genotype), [29]) “fish nodavirus”	Anti-VER K67 [32]
IPNV Sp, SK1433/09 field isolate Norway	Anti-IPN BIO 345, BIO-X Diagnostics
lumpfish flavivirus CLuV, field outbreak	na

## Data Availability

The data presented in this study are available on request from the corresponding author.
